# Sharing Individual Participant Data (IPD) within the Context of the Trial Reporting System (TRS)

**DOI:** 10.1371/journal.pmed.1001946

**Published:** 2016-01-19

**Authors:** Deborah A. Zarin, Tony Tse

**Affiliations:** National Library of Medicine, National Institutes of Health, Bethesda, Maryland, United States of America

## Abstract

Deborah Zarin and Tony Tse of ClinicalTrials.Gov consider how sharing individual participant data can and cannot help improve the reporting of clinical trials.

**Summary Points**

- The role of individual participant data (IPD) sharing can best be understood as part of an overall three-level trial reporting system (TRS) framework.
- Different “types” of IPD, which reflect varying degrees of information granularity, have different potential benefits and harms.
- Study 329 of Paxil (paroxetine) in children with depression is used as a case study to highlight the potential value of different components of the TRS.

The Institute of Medicine (IOM) [1], journal editors [2,3], and many others [4–6] have called for more widespread, third-party access to the individual participant data (IPD) and associated documentation from clinical trials (i.e., “IPD sharing”). Advocates assert that access to trial IPD will help to address well-established flaws in the current system of communicating trial results, including nonpublication, selective reporting, and lack of reproducibility [7]. Additional proposed benefits include the ability to reanalyze study data (e.g., validation and/or correction of previously published findings [8]) and to combine data from multiple studies (e.g., IPD-level meta-analyses [9]). Others note the burdens and costs associated with preparing IPD and associated documentation for sharing, the need to ensure participant privacy, and the risk of invalid analyses [10].

We do not attempt to replicate the more comprehensive analysis of IPD sharing that was conducted by the recent IOM panel [1]. However, we believe that it would be helpful at this pivotal time to consider the implications of IPD sharing within the context of the “trial reporting system” (TRS), which encompasses existing efforts to enhance access to information about trials and their findings and to improve the transparency of the clinical research enterprise (CRE) [11]. In this essay, we attempt to add precision to the ongoing discussion by examining the range of information granularity associated with different types of IPD. We then consider IPD sharing within a three-level TRS framework and illustrate the roles of these levels with a case study.

## What Is the Nature of IPD?

**Fig 1:**
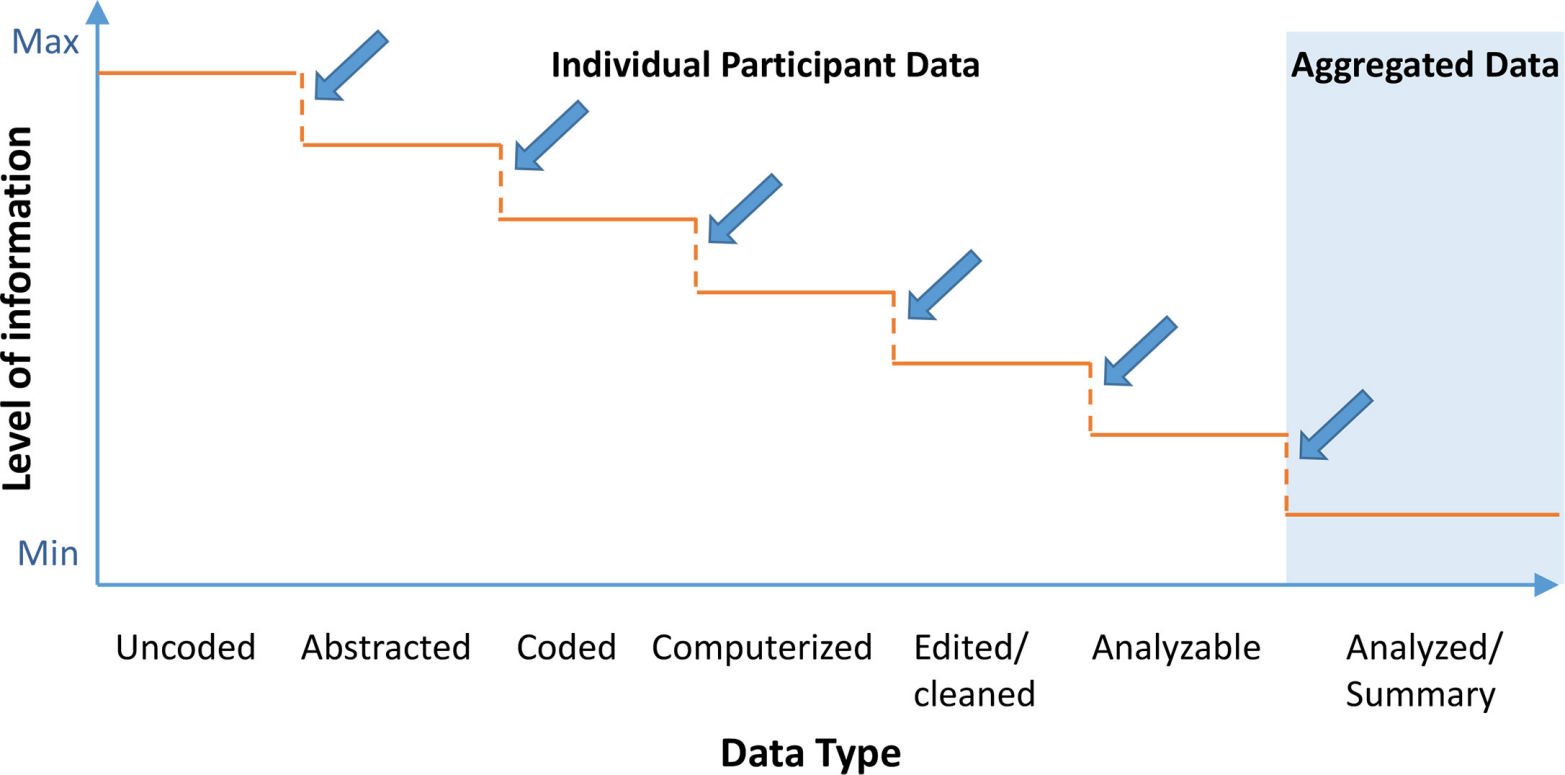
Schematic depicting information granularity for different types of data [12].

As attention shifts to IPD sharing, it is instructive to consider the mechanism by which initial “raw” data collected from each trial participant are analyzed, transformed, and aggregated into the summary data reported in the results sections of journal articles, conference abstracts, press releases, and package inserts and as entries in results databases (Fig 1).

Each arrow in Fig 1 indicates a transformation of trial data. While some transformations are based on procedures prespecified in study documents (e.g., detailed criteria or algorithms in the protocol or statistical analysis plan), others likely rely on ad hoc expert judgments. For example, analyzing IPD collected for the primary outcome measure of “change in tumor size from baseline at 3 months” might involve the following decisions:

- choosing a specific imaging approach (e.g., fluorodeoxyglucose (FDG)-positron emission tomography (PET) using a specific device);
- determining a particular method for transforming 2- or 3-D images into tumor size measurements (e.g., Digital Imaging and Communications in Medicine [DICOM] standard using autocontouring to calculate the volume for the region of interest);
- applying these methods to measure tumor size for each individual at baseline and at 3 months; and
- calculating and recording the changes in size per participant.

Additional decisions must be made by the researchers about the handling of missing data, unreadable images, and other data deficiencies; determining the analysis population (e.g., all who started the study [including those who discontinued] or only those who received the full course of treatment); and aggregating the IPD for purposes of reporting and analysis (e.g., mean change in size versus proportion with a change over a certain size). The most granular data (far left in Fig 1) would provide insight into these decisions and allow independent researchers to examine the implications of alternative analytic decisions. On the other hand, the least granular IPD (far right) would obscure some of these decisions and would not allow for testing the impact of different analytic methods.

Most discussions of IPD sharing policies sidestep the issue of matching IPD types with anticipated benefits and burdens. For example, third-party researchers interested in independently recoding the IPD would need access to uncoded data (i.e., data types to the left of “Coded” on the *x*-axis in Fig 1). In contrast, users who intend to replicate and confirm the reproducibility of aggregate data published in a journal article may only require access to the analyzable IPD (i.e., final type of IPD before undergoing transformation into aggregated data in Fig 1). While not an insurmountable barrier for IPD sharing policies, we believe that consideration of various data types and their uses is a timely issue for discussion within the research community, including questions such as the following:

- What standard terminology or classification should be used to describe the different data types?
- Which types of IPD should be made available systematically?
- When more than one type is available for sharing, how should they be uniquely identified and tracked (e.g., cited) within the research community?

## Where Does IPD Fit in the TRS?

**Fig 2:**
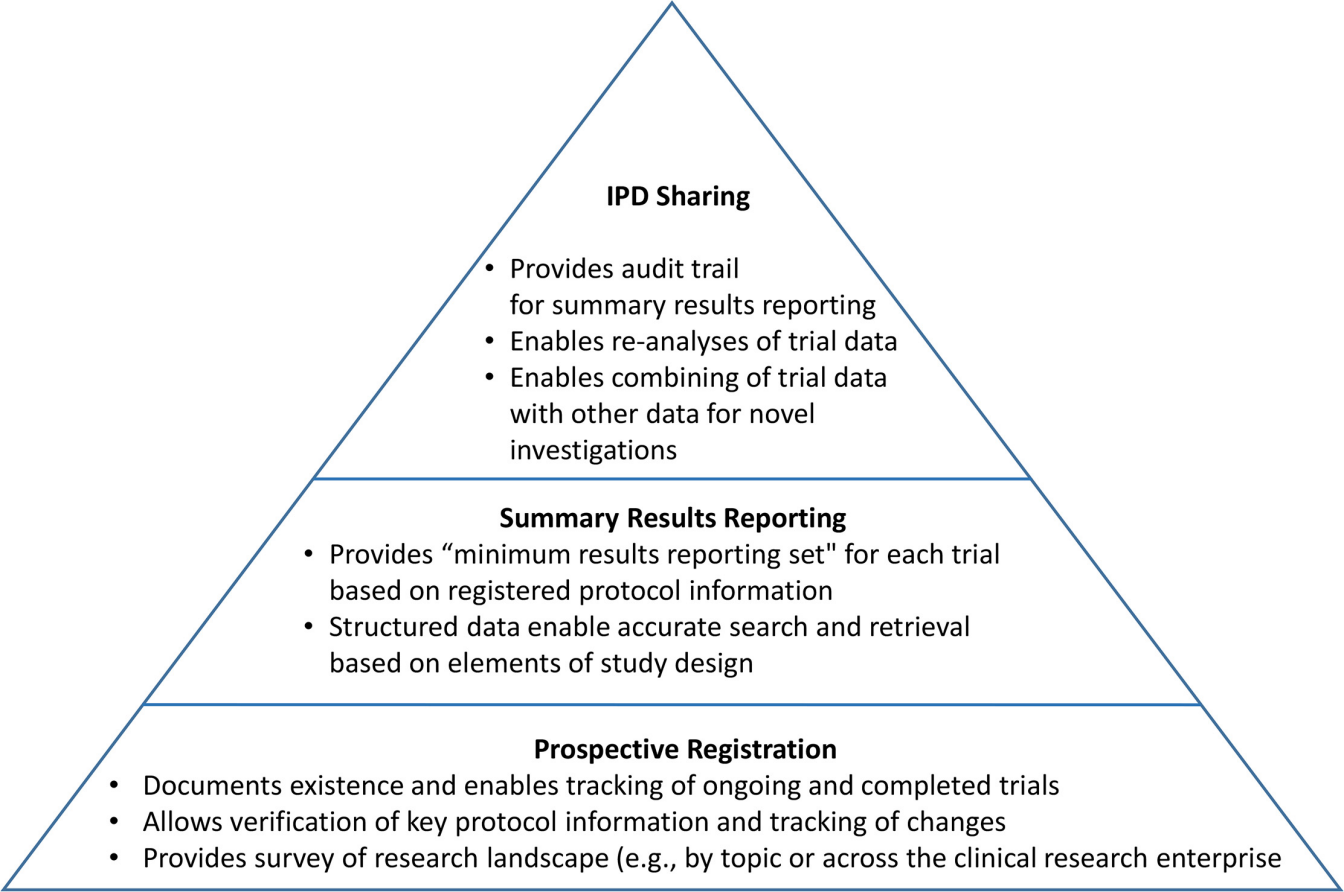
Schematic depicting the functions of the three key components of the TRS.

The TRS framework encompasses key existing and proposed efforts and is designed to increase trial transparency systematically. Fig 2 depicts the TRS as a pyramid with prospective registration at its base, summary or aggregate trial results reporting in the middle, and the sharing of trial IPD and relevant documents at its apex.

At its base, prospective registration provides a public listing of all ongoing and completed trials, along with key protocol and administrative details to allow people to identify the full set of trials conducted within a research area (e.g., antidepressant trials in children). Trial registration, if done and used appropriately, also allows for the assessment of fidelity to key protocol details, such as definition of the prespecified primary outcome measure [13]. Summary results reporting in trial registries, currently implemented at ClinicalTrials.gov and the European Union Clinical Trials Registry [14], is the next level of the TRS. Results databases—designed to ensure that aggregate trial results are reported systematically in a timely, structured, and complete manner based in part on expert trial-reporting guidelines such as the Consolidated Standards of Reporting Trials (CONSORT) statement [15] and its extensions—call attention to unacknowledged deviations from the registered protocol details [13]. Current policies are generally intended to address these two foundational levels of the TRS.

**Fig 3:**
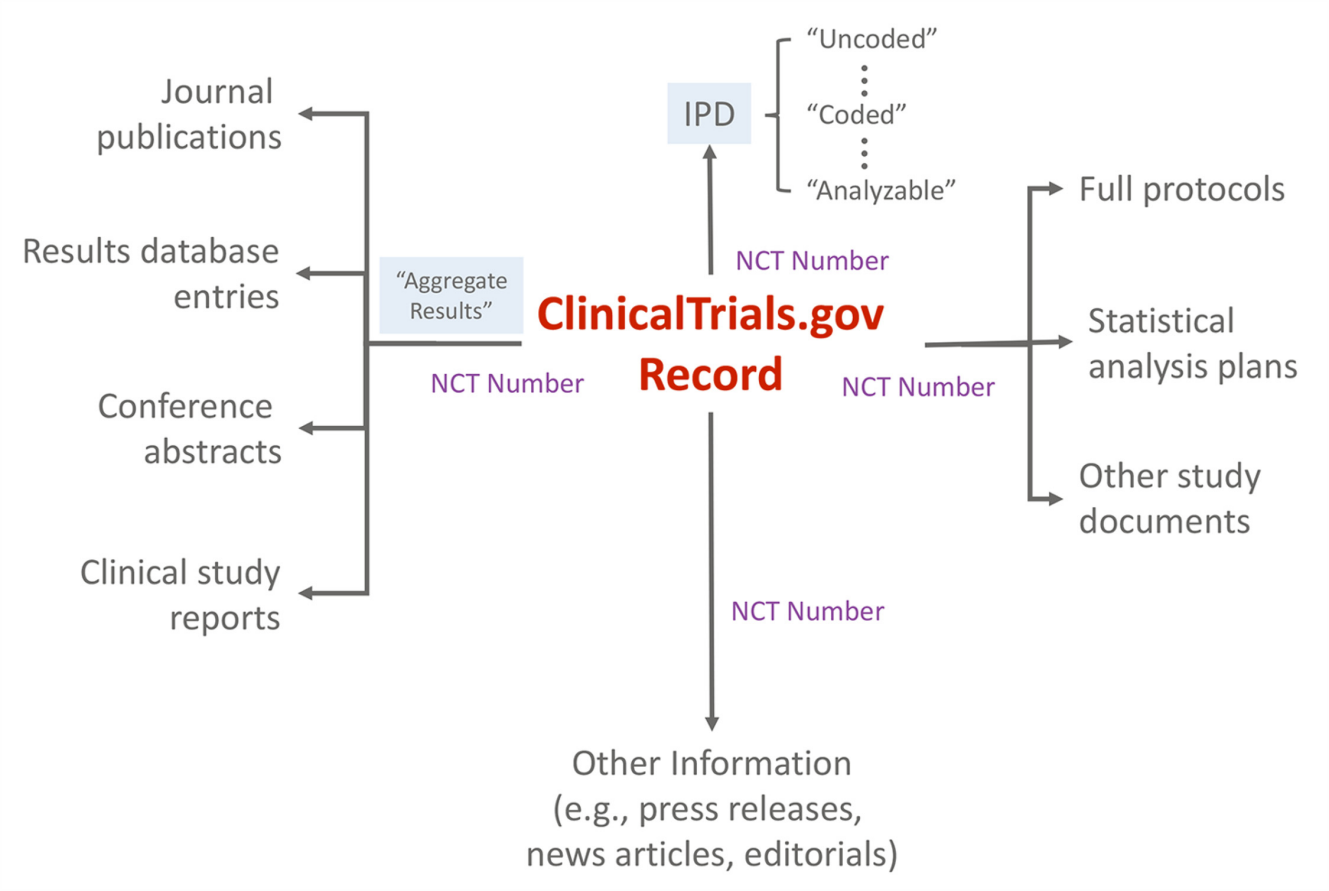
Schematic depicting ClinicalTrials.gov as an “information scaffold” using the record unique identifier (NCT number) to link to various online resources.

Registration information and summary results displayed as a single trial record provide the minimal, essential information needed to understand a trial and its findings. Each record also uses a format that is highly structured and searchable by a range of criteria. Ideally, users could easily retrieve information about all completed or ongoing trials for a particular clinical or policy question (e.g., to identify a need for additional research or conduct a systematic review), avoiding the biases imposed by incomplete and selective publication. Trial registration and results records are also linked, via unique registry identifiers, to relevant peer-reviewed journal publications [16]. As the use of unique registry identifiers expands (e.g., systematic reviews and press releases), an extensive network of automated, explicit linkages can provide an even more useful way to identify publicly available information about a trial from the trial record itself (Fig 3).

IPD and related documents reside at the apex of this pyramid because they are most useful within the context of the two lower levels, which serve as the foundation. Without careful use of trial registries and summary results databases, access to IPD might simply recreate or amplify existing reporting biases [17]. For example, analysis of trial IPD cannot mitigate biases that stem from selective release of data from only one trial among a “family” of trials for the studied population, intervention, and condition (e.g., a likely result of proposals to require the release of IPD only upon journal publication).

## How Would the Three Key Components of TRS Work Together?

### Case Study: Recent Reanalysis of Study 329

Study 329, sponsored by SmithKline Beecham (now GlaxoSmithKline [GSK]), was one of several studies conducted to examine the use of Paxil (paroxetine) in children with depression and the first with results to be published. The original publication of Study 329 in 2001 implied that the study results showed the safety and efficacy of Paxil in children [18]. In 2004, the New York State attorney general filed a consumer fraud lawsuit against GSK, alleging that the suppression and misreporting of trial data created the false impression that Paxil was safe and effective in depressed children [19].

A newly published reanalysis, part of the Restoring Invisible and Abandoned Trials (RIAT) initiative [20], was based on access to original case report forms (CRFs) for 34% of the 275 participants [21]. These highly granular IPD datasets enabled the researchers to recategorize certain adverse events that they determined had been miscategorized originally (e.g., “mood lability” rather than the more serious “suicidality”). The reanalysis concluded that Study 329 did not show either efficacy or safety.

### How Would the Problems of Study 329 Be Addressed by the Current TRS?

It would be an oversimplification to conclude that this reanalysis demonstrates the need to make IPD for all trials available. A more nuanced look at the specific problems is useful. Many of the concerns about Study 329 and the other Paxil studies might have been addressed if current policies regarding registration and results reporting had been in existence (Table 1, [22–24]). The key issue that specifically required access to IPD was the detection of miscategorization of some adverse events in the original report.

**Table 1:** Key issues with trials of antidepressant use in children for depression and the role of the TRS.

Key Issue	Relevant TRS Component	Comment
Lack of prospective public information about all trials of Paxil and other selective serotonin reuptake inhibitors (SSRIs) in depressed children	Prospective Registration	Registration would have provided a public list of all ongoing and completed trials of Paxil/SSRIs in depressed children
Alleged suppression of “negative” results from certain Paxil trials in depressed children [22]	Prospective Registration	Registration would have allowed the detection of trials without disclosed results
	Summary Results Reporting	Results database entries would have provided access to “minimum reporting set” including all prespecified outcome measures and all serious adverse events
Detection of selective reporting bias of efficacy and safety findings in the published results of Study 329, unacknowledged changes in outcome measures, and other issues [23]	Prospective Registration	Archival registration information would have allowed for the detection of unacknowledged changes in prespecified outcome measures and detection of nonprespecified outcome measures reported as statistically significant
	Summary Results Reporting	Structured reporting devoid of interpretation or conclusions would have made summary data publicly available while avoiding the possibility of spinning the results
Invalid and unacknowledged categorization of certain adverse events, resulting in the underreporting of suicidality [24]	Sharing Highly Granular IPD and Documents (e.g., CRFs)	Access to high-granularity IPD enabled the elucidation of data analytic decisions that had not been publicly disclosed; reanalysis was possible with different methods of categorizing adverse events

It is important to note that this illuminating reanalysis required access to the highly detailed IPD available in the original CRFs, represented by the far-left side of the *x*-axis in Fig 1. However, recent high-profile proposals for the sharing of IPD might not have added any clarity in the case of the Paxil studies in children beyond what could have been achieved with the optimal use of a registry and results database (i.e., two foundational levels of the pyramid in Fig 2). The reason is that journal publication serves as the “trigger” for IPD release in many of these proposals [1]), which could not possibly mitigate biases resulting from selective publication in the first place (i.e., IPD from unpublished trials would be exempt from sharing requirements). In addition, such proposed IPD policies call for the release of only the “coded” or “analyzable” dataset, which would not have allowed for the detection of miscategorization or the recategorization of the adverse events. Finally, such proposals would only require the sharing of a subset of IPD and documents for those aggregate data reported in the publication and not the full dataset, precluding secondary analyses intended to go beyond validation and reproducibility of the original publication.

## Conclusion

The evolving TRS can be thought of as a pyramid, with each successive layer being dependent on the layer(s) below it. We should not allow the prospects for providing access to IPD and relevant documents to divert attention from the continuing need to ensure complete, accurate, and timely trial registration and summary results reporting—as well as attentive and consistent use of these tools by key stakeholders. In addition, IPD sharing policies and systems must consider the different benefits and burdens that would be expected from third-party access to data types of varying levels of granularity.

## Abbreviations

CONSORT: Consolidated Standards of Reporting Trials
CRE: clinical research enterprise
CRF: case report form
DICOM: Digital Imaging and Communications in Medicine
FDG: fluorodeoxyglucose
GSK: GlaxoSmithKline
IOM: Institute of Medicine
IPD: individual participant data
NLM: National Library of Medicine
PET: positron emission tomography
RIAT: Restoring Invisible and Abandoned Trials
SSRI: selective serotonin reuptake inhibitor
TRS: trial reporting system

## References

[1] Institute of Medicine (2015) Sharing clinical trial data: maximizing benefits, minimizing risks Washington DC: The National Academies Press. 25590113

[2] Drazen JM (2015) Sharing individual patient data from clinical trials. N Engl J Med 372: 201–202. 10.1056/NEJMp1415160 25587944

[3] Data Availability Policy. PLoS Med; 2016 [cited 2016 Jan 6]. http://journals.plos.org/plosmedicine/s/data-availability#loc-acceptable-data-sharing-methods.

[4] Eichler HG, Abadie E, Breckenridge A, Leufkens H, Rasi G (2012) Open clinical trial data for all? A view from regulators. PLoS Med 9: e1001202 10.1371/journal.pmed.1001202 22505851 PMC3323505

[5] Krumholz HM, Peterson ED (2014) Open access to clinical trials data. JAMA 312: 1002–1003. 10.1001/jama.2014.9647 25203080

[6] Mello MM, Francer JK, Wilenzick M, Teden P, Bierer BE, et al (2013) Preparing for responsible sharing of clinical trial data. N Engl J Med 369: 1651–1658. 10.1056/NEJMhle1309073 24144394

[7] Song F, Parekh S, Hooper L, Loke YK, Ryder J, et al (2010) Dissemination and publication of research findings: an updated review of related biases. Health Technol Assess 14: iii, ix-xi, 1–193. 10.3310/hta14080 20181324

[8] Ebrahim S, Sohani ZN, Montoya L, Agarwal A, Thorlund K, et al (2014) Reanalyses of randomized clinical trial data. JAMA 312: 1024–1032. 10.1001/jama.2014.9646 25203082

[9] Tierney JF, Vale C, Riley R, Smith CT, Stewart L, et al (2015) Individual Participant Data (IPD) Meta-analyses of Randomised Controlled Trials: Guidance on Their Use. PLoS Med 12: e1001855 10.1371/journal.pmed.1001855 26196287 PMC4510878

[10] Tudur Smith C, Dwan K, Altman DG, Clarke M, Riley R, et al (2014) Sharing individual participant data from clinical trials: an opinion survey regarding the establishment of a central repository. PLoS ONE 9: e97886 10.1371/journal.pone.0097886 24874700 PMC4038514

[11] Zarin DA, Tse T (2008) Medicine. Moving toward transparency of clinical trials. Science 319: 1340–1342. 10.1126/science.1153632 18323436 PMC2396952

[12] Zarin DA (2012) Presentation at IOM Workshop on Sharing Clinical Research Data. Washington DC.

[13] Tse T, Zarin DA, Williams RJ, Ide NC (2012) The role and importance of clinical trial registries and results databases In: Gallin JI, Ognibene FP, editors. Principles and Practice of Clinical Research 3rd ed Amsterdam: Academic Press pp. 171–181.

[14] EU Clinical Trials Register. European Medicines Agency. [cited 2016 Jan 6] https://www.clinicaltrialsregister.eu/ctr-search/search.

[15] Schulz KF, Altman DG, Moher D, Group C (2010) CONSORT 2010 statement: updated guidelines for reporting parallel group randomised trials. PLoS Med 7: e1000251 10.1371/journal.pmed.1000251 20352064 PMC2844794

[16] Clinical Trial Registry Numbers in MEDLINE^®^/PubMed^®^ Records. U.S. National Library of Medicine. [cited 2016 Jan 6] https://www.nlm.nih.gov/bsd/policy/clin_trials.html.

[17] Zarin DA (2013) Participant-level data and the new frontier in trial transparency. N Engl J Med 369: 468–469. 10.1056/NEJMe1307268 23902488

[18] Keller MB, Ryan ND, Strober M, Klein RG, Kutcher SP, et al (2001) Efficacy of paroxetine in the treatment of adolescent major depression: a randomized, controlled trial. J Am Acad Child Adolesc Psychiatry 40: 762–772. 11437014 10.1097/00004583-200107000-00010

[19] The People of the State of New York v. GlaxoSmithKline, Complaint, filed 2 June 2004. [cited 2016 Jan 6] http://news.findlaw.com/wsj/docs/glaxo/nyagglaxo60204cmp.pdf.

[20] Doshi P, Dickersin K, Healy D, Vedula SS, Jefferson T (2013) Restoring invisible and abandoned trials: a call for people to publish the findings. BMJ 346: f2865 10.1136/bmj.f2865 23766480 PMC3685516

[21] Le Noury J, Nardo JM, Healy D, Jureidini J, Raven M, et al (2015) Restoring Study 329: efficacy and harms of paroxetine and imipramine in treatment of major depression in adolescence. BMJ 351: h4320 10.1136/bmj.h4320 26376805 PMC4572084

[22] GSK Assurance of Discontinuance, 2004. [cited 2016 Jan 6] http://www.ag.ny.gov/sites/default/files/press-releases/archived/aug26a_04_attach2.pdf.

[23] Jureidini JN, McHenry LB, Mansfield PR (2008) Clinical trials and drug promotion: Selective reporting of study 329. International Journal of Risk & Safety in Medicine 20: 76–81.

[24] Leslie LK, Newman TB, Chesney PJ, Perrin JM (2005) The Food and Drug Administration's deliberations on antidepressant use in pediatric patients. Pediatrics 116: 195–204. 15995053 10.1542/peds.2005-0074 PMC1550709

